# Wellbeing, social media addiction and coping strategies among Chilean adolescents during the pandemic

**DOI:** 10.3389/fpsyt.2023.1211431

**Published:** 2023-08-29

**Authors:** Jorge J. Varela, Janet Carola Pérez, Matías E. Rodríguez-Rivas, María Josefina Chuecas, Javiera Romo

**Affiliations:** Facultad de Psicología, Universidad del Desarrollo, Concepción, Chile

**Keywords:** wellbeing, life satisfaction, social media addiction, adolescents, coping, COVID-19

## Abstract

**Introduction:**

During the COVID-19 pandemic, adolescents had to deal with a range of mental health problems that has increased social media addiction levels with adverse effects on life satisfaction. Previous studies have explored coping mechanisms to deal with this addiction problem, but did not consider the need to simultaneously cope with different dimensions. Therefore, our study aimed to examine the moderating effect of various coping mechanisms on the relationship between social media addiction and adolescent life satisfaction.

**Methods:**

Self-report questionnaires were applied to 1290 secondary school students (age mean = 16.03, *SD* = 1.27, range: 14 to 19; and 57% female). An exploratory and a confirmatory factor analysis were performed to determine the factor structure of the Brief-Cope 28 scale. Then, a descriptive and correlational analysis of the variables and a multiple linear regression analysis was performed.

**Results:**

We found that the social media addiction risk was negatively associated with life satisfaction, adaptive strategies were positively correlated to life satisfaction, and maladaptive strategies were negatively correlated to it. Also, a moderation model was evaluated in which four stress management strategies, namely acceptance and perspective-taking, seeking socio-emotional support, active coping, and maladaptive strategies all conditioned the relationship between social media addiction risk and life satisfaction after controlling for demographic variables and the specific strategies of using comedy, religion and substance use. Results indicate additive and multiplicative effects of management strategies for stressful situations in the studied relationship. Seeking socio-emotional support and active coping were positively related to life satisfaction and maladaptive strategies were negatively associated with it. Multiplicative effects indicate that the relationship between the social media addiction risk and life satisfaction depends only on the acceptance and perspective taking that adolescents report. When adolescents reported having low or average levels of acceptance and perspective taking, there was a negative correlation with general life satisfaction, a connection that grew markedly stronger. In contrast, no connection between social media addiction and life satisfaction was detected for adolescents who report higher levels of acceptance and perspective-taking.

**Discussion:**

Abuse of social media and the use of maladaptive stress coping strategies were risk factors that decreased life satisfaction among adolescents during the COVID-19 pandemic period.

## Introduction

Wellbeing is an important aspect of mental health among adolescents. Yet, during the pandemic they had to face new risk factors with negative consequences, especially for their mental health. One risk factor, due the increasing need to connect to others online, was the heightened level of social media addiction among adolescents. Increased levels of social addiction can have negative consequences in the short and long term for mental health, which became more negative during the pandemic, considering the long hours spent online during this period. In such a context, recognizing individual capacities for handling negative consequences is vital for prevention among adolescents, including developing coping strategies to deal with stress. Even though previous studies have recognized the importance of coping strategies for adolescents’ mental health, they have not examined the possible protective effect coping can have, attenuating the negative effect of social media addiction on adolescent wellbeing. Therefore, the purpose of the study was to examine this relationship with Chilean adolescents during the pandemic.

### Social media addiction, wellbeing, and life satisfaction among adolescents

Subjective wellbeing is increasingly being considered as a central component of mental health and a determining element for a positive life for children and adolescents, as a key component of positive psychology framework (1). In turn, life satisfaction is a global cognitive evaluation that a person makes of his or her quality of life. It is also one of the key components of subjective wellbeing (2, 3), being different but highly correlated constructs.

In general, various countries around the world report high levels of wellbeing and life satisfaction during childhood and adolescence, based on a positive psychology framework. For example, one study that included data from 128,000 children between 8 and 12 years of age from 35 different countries found that they reported high satisfaction with their lives, especially in the life domains related to family and home (4). Similar results have been found in Chile, also reporting high levels of wellbeing and life satisfaction in this population (5). During the pandemic, however, life satisfaction levels significantly decreased (6).

For example, globally, an umbrella review that included 17 systematic reviews of studies on the effect of the pandemic on adolescent mental health found that adolescents had higher levels of depression, anxiety, sleep disorders, stress-and trauma-related disorders, suicidal ideation, attention disorders, and general mental health problems compared to levels before the onset of the pandemic (7), negatively affecting wellbeing and life satisfaction. Similarly, a study conducted in Chile that evaluated satisfaction with life among other variables and the experiences of adolescents during the pandemic period reported that adolescent mental health was disturbed, introducing higher levels of worry, fear and nervousness in this group with lower levels of energy, happiness and confidence compared to the time before the COVID-19 pandemic (8).

Studies have shown that life satisfaction has been strongly associated with mental health self-report (9). Specifically, life satisfaction during adolescence has been linked to variables such as self-esteem, perceived social support, emotional stability, interpersonal competencies among others (10, 11).

Moreover, factors such as substance abuse, aggressive behaviors, poor parental relationships, increased victimization, depressive mood, and social media addiction and several others have been found to function as risk factors for life satisfaction of children and adolescents (6, 12–14).

Specifically, social media addiction has been found to constitute an important risk factor for adolescents’ life satisfaction (15). Despite advances in understanding the relationship between life satisfaction and social media addiction during adolescence, there is still a lack of data to clarify this relationship in the context of the pandemic.

### Social media addiction among adolescents during the pandemic

The WHO formally declared the COVID-19 infection to be epidemic around the world in 2020 (16), which had negative effects for adolescents worldwide (17). During this period, internet use increased significantly with possible negative effects on adolescents. For example, Marciano and colleagues (18) conducted a systematic review of 30 studies that had been published up to September 2021 about the relationship between the mental health and digital media use of adolescents during COVID-19, demonstrating a positive correlation with media addiction and ill-being. Likewise, a review regarding the effect of social media use on mental health during the pandemic in the adolescent population reported an increased use of screens during the first year of the pandemic, which negatively impacted adolescent mental health (19).

Today’s adolescents are known as “digital natives” as they have been exposed to the use of digital platforms and related technologies from an early age (18). Social networks, mobile messaging, and microblogging services like Twitter, Tik Tok, Mastodon, Facebook, WhatsApp, Messenger, Instagram, YouTube, LinkedIn, Reddit, Snapchat, Amino, Signal, Telegram, WeChat and others are established information media, especially among adolescents (20). At a younger age, platforms are used for social and entertainment purposes. As age increases, they begin to get used for accessing information and educational material (21). Adolescents use these media as one of their primary ways of interacting and directly communicating with other people, generating a sense of belonging in their social lives (22).

Social network use has grown steadily worldwide, which saw a significant jump during the pandemic, growing by 13% between January 2020 and January 2021 with 490 million new user accounts (23, 24). In Chile, approximately 17.85 million users are active on social networks, representing 92.8% of the national population. The number stands at 227 million worldwide after growing by 5% in 2022, equivalent to 59% of the world population (24). When we look at adolescents in Chile, we see that 88% have a fixed broadband connection at home and 81% have a cell phone. Due to such widespread use, more than 50% acknowledge that they have less time for engaging in other activities (studying, playing sports, spending time with family) because of being online for so long; 58% for those under 12 years of age and 54% for above 12 years of age.

Despite all the benefits related to using the Internet and social media, especially during the pandemic period, since it frequently enabled the continuation of classes or meetings and facilitated contact with family and friends, the indiscriminate and unsupervised use of this technological tool also brought negative consequences. According to the literature, the negative influence of the Internet and social media on mental health and specifically on life satisfaction had been reported even before the onset of the pandemic; however, during this period its influence grew even more significant (19). Specifically, a peer review that looked at the effects of the pandemic on adolescent mental health reported that one of the negative consequences associated with indiscriminate internet use is social media addiction, among other consequences (7).

Social media addiction refers to the excessive and uncontrollable need to get online and is one of the most worrying current public health issues, especially among adolescents (25). In the last decade several terms have been used to describe this behavior such as “compulsive computer use,” “internet dependence,” “pathological internet use,” and “internet addiction” (26). Studies prior to the pandemic had placed the prevalence of social media addiction in ranges varying from 3 to 17% (27, 28). In contrast, studies conducted during or after the COVID-19 pandemic period report increased prevalence, ranging from 19 to 22% (25, 29). Therefore, more studies exploring the effect of social media addiction in the recent past are needed.

Although the concept of social media addiction was not included in the DSM-5 as a disorder, social media addiction is a global and widely-recognized problem (30). There are both emotional and physical characteristics of social media addiction, including mental health problems, lack of patience, isolation, disruption of social relationships, weight loss or weight gain, vision problems, poor nutrition, insomnia, and more (31). Numerous risk factors for social media addiction have been associated at the individual, family, relational, school and community levels (32, 33), reporting specific risk factors such as impulsivity, neuroticism, depression, anxiety, low family support, family dysfunction, and more.

Despite the numerous studies that have been conducted to better understand social media addiction, few have tried to understand the factors that protect against social media addiction. For example, a meta-analysis of 153 studies identified 56 risk factors for social media addiction and only 28 protective ones (32). Although progress has been made around identifying protective factors associated with coping strategies used by adolescents, not enough is known given the fact there are multiple types of strategies that can be both adaptive and maladaptive and may interact and relate differently when it comes to social media addiction (34, 35).

### Coping mechanisms as protective factors

Coping strategies are understood as complex cognitive, emotional and behavioral mechanisms that people use to cope with various adverse and stressful situations in order to reduce the negative consequences (36). These types of skills are of vital importance due to their effects on the interpersonal, family and psychosocial functioning of the population, especially during adolescence since this is a critical development period (37).

Several studies show that coping strategies are a fundamental resource for stress management situations and various difficulties, since the strategies determine the degree of problem resolution and their effects on quality of life and life satisfaction (38). In addition, we see that adaptive coping is associated with better mental health and life satisfaction outcomes, as well as lower levels of perceived stress, being a key resource for the optimal functioning of individuals in the face of adverse and highly stressful situations (39).

Previous studies show that adaptive coping strategies are a key resource as a protective factor of life satisfaction against the negative effects of perceived stress in adult and adolescent populations (40). We see also that when dealing with addiction, cognitive coping focused on problem-solving is a protective factor for mental health as well as for positive interpersonal relationships and reducing addictive symptoms (39, 40). Moreover, we see that maladaptive coping skills like denial and self-incrimination are risk factors that increase the risk of depression and suicide while reducing levels of life satisfaction in the population with addiction problems (41).

The COVID-19 pandemic period showed that coping strategies are a key resource for managing its negative effects; however, due to the prolonged effect and variability of coping strategies, many negative effects on wellbeing and an increased risk of addiction were detected in the population (42). Thus, cognitive and problem-focused coping strategies have been found to mitigate the negative impact of pandemic-related stressors on adolescents’ wellbeing levels (42). Nevertheless, there is little evidence on coping strategies and social network addiction in the adolescent population from the pandemic period.

Therefore, based on previous revision, we recognize that life satisfaction can be affected by social media addiction as a significant risk factor for adolescent mental health. Yet, we can recognize individual resources that can moderate this negative effect by becoming protective factors. Therefore, the study’s aims were: (1) To examine the effect of social media addiction on adolescent wellbeing. (2) Examine the positive effect of coping mechanisms on adolescent life satisfaction. (3) Test the interaction effect of coping mechanisms on the relationship between social media addiction on life satisfaction. Based on a prior review, we hypothesized that internet addiction would be negatively correlated with adolescent life satisfaction. We also hypothesized that coping mechanisms would have a moderating effect on this connection. More specifically, more and better coping mechanisms would ameliorate the negative effects of internet addiction on adolescent life satisfaction.

## Methodology

### Sample

A total of 1,320 students participated in the study; 57% were female (*n* = 753) aged 14–19 (*M* = 16.03 years, *SD* = 1.27). The students were in secondary school (grades 9–12), including nine private and public schools in three regions of Chile (Santiago the Metropolitan, Los Rios, and Bio Bio Regions). Schools were selected through convenience sampling.

### Data collection

The self-report questionnaire was applied online from April to July 2021, when major mobility restrictions and population confinement measures were implemented. In addition, during this period school children had to attend classes online due to the COVID-19 pandemic and school closures ordered by the Chilean Government. Data production was carried out by coordinating with each educational establishment, having first sent passive informed consent forms to the instructors and informed assent forms to the students.

Each data collection activity averaged 45 min in duration and was carried out by the research team during regular school hours by sending the questionnaire access link available on the Alchemer Survey platform. The project was approved by the Ethics Committee of the Universidad del Desarrollo de Chile. After obtaining informed consent from the teachers, students were required to sign an informed assent form before participating. They were not given any incentives for their participation.

### Instruments

#### Student life satisfaction scale

This instrument was developed by Huebner (43) for measuring global satisfaction with life in children and adolescents aged 8 to 18 years. This scale has been validated in Chile and shown to have adequate psychometric properties in Chilean children and adolescents (43, 44). An abbreviated four-item version was used for the present study, which assesses several measures of life satisfaction through an 11-point Likert-type response scale (0 = Strongly disagree; 10 = Strongly agree), which includes the following statements: “My life is going well,” “My life is the way I want it to be,” “I have a good life” and “I have what I want in life.” Higher scores on the scale indicate higher levels of life satisfaction. The reliability coefficient for our study’s sample was *a* = 0.902.

#### The Bergen social media addiction scale

The Bergen social media addiction scale (BSMAS) is a six-item instrument developed by Andreassen et al. (45) to assess the risk of social network addiction, adapted from the Bergen Facebook Addiction Scale (BFAS). It was translated into Spanish using translation and back-translation methodology (46). The scale was developed based on the six core components of addiction proposed by Kircaburun et al. (21) (i.e., salience, mood modification, tolerance, withdrawal conflict, and relapse). The instrument uses a five-point Likert-type scale ranging from very rarely (1) to very often (5). A higher score on the BSMAS is associated with a higher risk of social network addiction. Item examples: “How often during the last year have you felt an increasing need to use social networks?”; “Have you tried to stop using social networks and were not able to?”; “Have you used social networks so much that their use has negatively affected your work/studies?” The unidimensionality and satisfactory psychometric properties of the BSMAS have been confirmed in different languages in diverse populations (45, 46), including English, Italian, Persian, and Portuguese. The higher the score, the higher the self-report of addiction levels. The reliability coefficient for our study’s sample was *a* = 0.813.

#### The brief coping orientation to problems experienced (Brief-COPE-28)

The Brief-COPE was designed to measure effective and ineffective ways of coping with a stressful life event. It was developed as a short version of the original 60-item COPE scale (47), which was theoretically derived based on various models of coping. Brief-COPE is a 28 item self-report questionnaire that uses a 4-point Likert-type response scale to evaluate the frequency of various strategies used in stressful situations (1 = I usually do not do this at all, 4 = I usually do this a lot). Higher scores on each subscale indicate higher frequency of strategy use. The abbreviated version of 28 items has been validated in different languages and contexts, demonstrating different factorial structures, ranging from the grouping of 2 factors (adaptive and maladaptive strategies) to the structure proposed by the original English scale of 14 factors: active coping, planning, positive reframing, acceptance, humor, religion, having emotional support, having instrumental support, self-distraction, denial, venting, substance abuse, behavioral disengagement, and self-blame (47). For the present study we used the abbreviated version translated into Spanish by Morán et al. (48). In the Morán et al. study, an overall Cronbach’s alpha of 0.86 was obtained, and its factors ranged from 0.30 (acceptance) to 0.93 (substance use).

Given that one can find different factor structures and psychometric properties in the Brief COPE-28 scale depending on the selected population (49), coupled with the lack of information regarding the adolescent population, the factor structure of the Spanish version was analyzed with the Chilean adolescent sample before performing the analyses at the heart of this study. For this purpose, the data was divided into two random samples; one for exploratory factor analysis (EFA) (*n* = 660) and the other for confirmatory factor analysis (CFA) (*n* = 660). The EFA was estimated by the weighted generalized least squares (GLS) factorization method and oblimin rotation to account for non-normal distribution of items. This suggested a solution of 7-factors, including 26 items (2 items inverted). Absolute values of standardized factor loadings were between 0.35 and 0.93 (factor loadings range for each factor are presented in Supplementary Table S1).

The factors were: (a) socio-emotional support, considering emotional and instrumental support (4 items, i.e., “I’ve been getting emotional support from others”), (b) active coping, including thinking about how to cope with the stressor, planning, performing actions to eliminate or reduce the stressor, and negative behavioral disengagement items were included as reversed items (6 items, i.e., “I’ve been thinking hard about what steps to take”), (c) maladaptive strategies, includes self-blame, venting and denial strategies (5 items, i.e., “I’ve been criticizing myself”), (d) substance use, drinking alcohol or using other substances in order to feel good or to cope with the stressor (2 items, i.e., “I’ve been using alcohol or other drugs to help me get through it”), (e) humor (2 items, i.e., “I’ve been making jokes about it”), (f) religion or spiritual beliefs (2 items, i.e., “I’ve been praying or meditating”), (g) acceptance and perspective change, including items of acceptance, positive reframing, and self-distraction (5 items, i.e., “I’ve been trying to find the good in what is happening”).

This structure was confirmed in CFA with acceptable model fit values [*x*^2^(277) = 707.619, *p* < 0.001, CFI = 0.91, TLI = 0.90, SRMR = 0.05, RMSEA = 0.05 (90% CI, 0.046–0.055)]. This was estimated by the weighted least squares method adjusted for mean and variance (WLSMV) to account for non-normal distribution of items. Good fit was indicated by a nonsignificant chi-square, RMSEA and SRMR values less than 0.05. CFI and TLI greater than or equal to 0.95 are indicators of good fit and between 0.90 and 0.95 as acceptable values (for more information on the factor analyses, see Supplementary material).

The overall reliability of the present study, as assessed by Cronbach’s alpha, was *a = 0*.77, and the alphas of the scales ranged from *a* = 0.60 (acceptance and change of perspective scale) to *a = 0*.91 (substance abuse scale).

#### Demographic variables

The age (continuous variable) and gender (dichotomous variable; 0 = Male and 1 = Female) variables were used as self-reported control variables.

### Data analysis

Initially, an exploratory factor analysis and a confirmatory factor analysis were performed to determine the factor structure of the Brief-Cope 28 scale in the Chilean adolescent population. The RStudio program version 4.1.3 was used for this purpose.

A descriptive and correlational analysis of the variables was carried out. For this, and the following analyses, the total mean score of the student life satisfaction scale (SLSS), the total mean score of the BSMAS scale and mean scores of the 7 factors of the COPE scale were used. In addition, demographic variables (age and gender) as control variables were used.

To account for the study objective, a multiple linear regression analysis was performed to examine the additive and multiplicative effects of social media addiction risk (BSMAS scale) and coping strategies (COPE scale) on students’ life satisfaction (SLSS scale). The hierarchical linear regression analysis was performed by entering the predictor variables of the model in the following order: gender and age (first step), social media addiction risk (second step), adaptive and maladaptive coping strategies (third step), and 4 two-way interaction terms of the most important coping strategies (acceptance and change of perspective, socio-emotional support, active coping and maladaptive strategies) for social media addiction risk were included at the end (fourth step). The quantitative variables of the model were mean-centered. Mean-centered variables were used to fit the interaction terms (50).

Two procedures were used to interpret significant interaction terms. The first was the simple slopes method (50, 51) in which a few values of the moderator are chosen to be fixed (usually mean value, one SD plus the mean and one SD less the mean), and the significance of the focal predictor of the criterion variable is estimated. The main drawbacks of this method are that any value for the moderator can be considered arbitrary (52). To overcome this weakness, the Johnson and Neyman (JN) technique (52) was used. This technique solves for the moderator values for which the effect of the predictor on the criterion variable becomes or ceases to be significant by estimating the lower and upper bounds of the confidence bands, estimating the effect of a predictor on the criterion variable. Also, a graph of the confidence bands makes it easy to show for which values of the moderator the effect of the focal predictor on the criterion variable is significant (52).

Descriptive and multiple regression analyses were performed with the SPSS version 26. Also, the PROCESS macro version 3.5 for SPSS (51) was used to estimate simple slopes and for the JN technique. Since the PROCESS macro does not have the option of including 4 two-way interactions, the simple interaction model was used instead (model 1), with acceptance and perspective change as the moderator in the relationship of the focal predictor relationship (risk for social media addiction) on students’ life satisfaction, controlling for the other variables included in the model (sex, age, other coping strategies and the remaining 3 two-way interactions). The graphs were created in Excel.

## Results

### Descriptive

Bivariate correlations, means, and standard deviations for demographic variables, social media addiction risk, coping strategies and students’ life satisfaction are presented in Table 1. The social media addiction risk was negatively associated with students’ life satisfaction (*r* = −0.28, *p* < 0.001). With respect to coping strategies, most adaptive ones were positively related to general life satisfaction (*r* = 0.09–0.39) and maladaptive strategies were inversely correlated with it (*r* = −0.18 to −0.42). Only the use of humor did not follow the aforementioned pattern, presenting a negative correlation with life satisfaction (*r* = −0.08, *p* < 0.01). Moreover, only active coping (*r* = −0.18, *p* < 0.001), but not the other adaptive strategies, was negatively correlated to social media addiction risk, and maladaptive ones were directly correlated with it (*r* = 0.14–0.37). In this case, both humor and religion use for coping with stress were associated with a higher social media addiction risk.

**Table 1:** Descriptive statistics and correlation for study variables.

Variable	*n*	*M*	*SD*	1	2	3	4	5	6	7	8	9	10	11
1. Sex	1,320	0.57	0.50	–										
2. Age	1,320	16.03	1.27	−0.01	–									
3. Life Satisfaction	1,320	6.75	2.12	−0.28^***^	0.01	–								
4. Internet addiction	1,263	2.43	0.85	0.34^***^	0.05	−0.28^***^	–							
5. Active coping strategies	1,300	2.92	0.53	−0.16^***^	0.09^***^	0.39^***^	−0.18^***^	–						
6. Maladaptive strategies	1,300	2.06	0.57	0.23^***^	−0.02	−0.42^***^	0.37^***^	−0.25^***^	–					
7. Substance use strategies	1,300	1.15	0.46	0.06*	0.10^***^	−0.18^***^	0.14^***^	−0.15^***^	0.18^***^	–				
8. Humor strategies	1,298	2.46	0.97	0.05	−0.01	−0.08^**^	0.13^***^	−0.01	0.21^***^	0.10^***^	–			
9. Religion strategies	1,287	1.58	0.77	0.06^*^	0.02	0.10^***^	0.09^***^	0.18^***^	0.04	−0.05	−0.05	–		
10. Acceptance and change of perspective strategies	1,300	2.60	0.55	0.00	0.03	0.17^***^	0.05	0.29^***^	0.05	−0.05	0.22^***^	0.21^***^	–	
11. Socio-emotional support strategies	1,300	2.30	0.71	−0.16	0.03	0.27^***^	0.04	0.25^***^	0.03	−0.06^*^	0.05	0.19^***^	0.26^***^	–

Pairwise deletion was applied to correlations.

^*^p < 0.05, ^**^p < 0.01, ^***^p < 0.001.

### Additive effects of social media addiction risk and coping strategies on students’ life satisfaction

First, we examined the social media addiction risk and coping strategies as predictors of students’ life satisfaction, controlling for participants’ age and gender with a hierarchical linear regression model (steps 1–3, Table 2).

**Table 2:** Hierarchical regression results for adolescent’s life satisfaction.

Variable	*B*	95% CI for B		*SE* B	*β*	*R* ^2^	∆*R*^2^
		LL	UL				
Model 1						0.08	0.08^***^
Constant	7.44^***^	7.27	7.64	0.09			
Sex	−1.22^***^	−1.45	−0.99	0.12	−0.28^***^		
Age	0.04	−0.05	0.13	0.05	0.02		
Model 2						0.12	0.04^***^
Constant	7.27^***^	7.09	7.44	0.09			
Sex	−0.92^***^	−1.16	−0.68	0.12	−0.21^***^		
Age	0.06	−0.03	0.15	0.05	0.04		
Internet addiction	−0.51^***^	−0.65	−0.38	0.07	−0.21^***^		
Model 3						0.35	0.23^***^
Constant	7.10^***^	6.95	7.25	0.08			
Sex	−0.62^***^	−0.83	−0.42	0.11	−0.15^***^		
Age	0.00	−0.07	0.07	0.04	0.00		
Internet addiction	−0.21^**^	−0.34	−0.09	0.07	−0.08^**^		
Active coping^a^	0.78^***^	0.57	0.98	0.10	0.19^***^		
Acceptance and change of perspective^a^	0.32^**^	0.13	0.52	0.10	0.08^**^		
Socioemotional support^a^	0.60^***^	0.45	0.74	0.07	0.20***		
Maladaptive strategies^a^	−1.15^***^	−1.34	−0.95	0.10	−0.03^***^		
Substance use^a^	−0.26^*^	−0.48	−0.04	0.11	−0.05^*^		
Humor^a^	−0.04	−0.14	0.07	0.05	−0.02		
Religion^a^	0.10	−0.03	0.23	0.07	0.04		
Model 4						0.35	0.01^***^
Constant	7.08^***^	6.92	7.24	0.08			
Sex	−0.60^***^	−0.81	−0.40	0.11	−0.14^***^		
Age	0.01	−0.07	0.08	0.04	0.00		
Internet Addiction	−0.22^**^	−0.35	−0.09	0.07	−0.09^**^		
Active coping^a^	0.77^***^	0.57	0.97	0.10	0.19^***^		
Acceptance and change of perspective^a^	0.31^**^	0.11	0.50	0.10	0.08^**^		
Socioemotional support^a^	0.62^***^	0.48	0.76	0.07	0.21^***^		
Maladaptive strategies^a^	−1.14^***^	−1.33	−0.95	0.10	−0,3^***^		
Substance use^a^	−0.25^*^	−0.47	−0.03	0.11	−0.05^*^		
Humor^a^	−0.04	−0.15	0.06	0.05	−0.02		
Religion^a^	0.09	−0.04	0.22	0.07	0.03		
Internet addiction * Active coping^a^	0.17	−0.06	0.40	0.11	0.04		
Internet addiction * Acceptance and change of perspective^a^	0.31^**^	0.10	0.52	0.11	0.07^**^		
Internet addiction* Socioemotional support^a^	−0.14	−0.31	0.03	0.09	−0.04		
Internet addiction* Maladaptive strategies^a^	0.11	−0.08	0.31	0.10	0.03		

CI, confidence interval; LL, Lower limit; UL, Upper limit. ^a^Stress-coping strategies.

^*^p < 0.05, ^**^p < 0.01, ^***^p < 0.001.

The first step of the hierarchical multiple regression indicated that control variables accounted for 8% of the life satisfaction variance, *F*(2,1257) = 55.91, *p* < 0.001. In this step, coefficients indicated that females had a lower level of life satisfaction than males and that the students’ age was unrelated. Furthermore, the second step indicated that while controlling for demographic variables, social media risk was negatively associated with life satisfaction, accounting for the 3.7% of life satisfaction variance, *F*(1.1256) = 53.33, *p* < 0.001. The third step indicated that, controlling for students’ age and sex, both social media risk (*β* = −0.09, *p* < 0.001) and some stress coping strategies were significant predictors of student’ life satisfaction. This model accounted for 35% of the life satisfaction variance, *F*(10.1249) = 68.10, *p* < 0.001. Specifically, active coping, acceptance and perspective change and socio-emotional support were positively correlated to student’ life satisfaction, with substance use and maladaptive strategies being negatively correlated.

### Multiplicative effects of stress coping strategies in the connection between social media addiction risk with students’ life satisfaction

To clarify the moderating role of coping strategies in the association of social media risk and students’ life satisfaction, in step 4 of the regression model (Table 2) we included a series of two-way interaction terms related to four specific coping strategies (acceptance and perspective change, socio-emotional support, active coping and maladaptive strategies) and social media addiction risk variables.

This last step of the hierarchical multiple regression indicated that interaction terms accounted for less than 1% of the life satisfaction variance, *F*(4, 1,245) = 3.59, *p* < 0.01. In this model, coefficients indicated that the acceptance and perspective change coping strategy is able to modify the negative relationship between social media addiction risk and student’ life satisfaction. No other interaction term was statistically significant.

**Figure 1:**
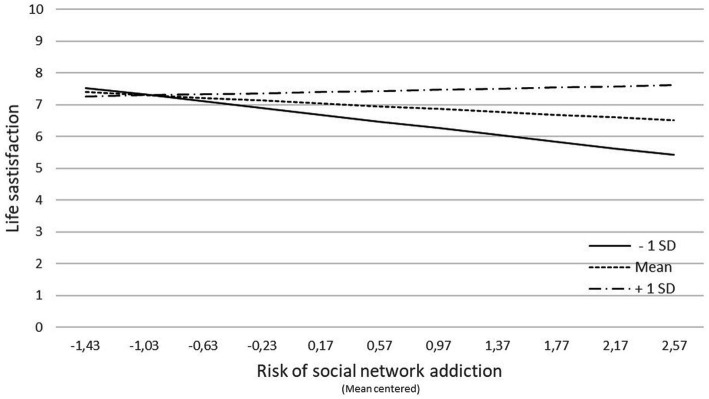
Moderating effect of acceptance and perspective change coping strategy on the relationship of risk of social network addiction to student’ life satisfaction: Simple slopes at 1 SD below the mean (− 1 SD), mean value (Mean) and 1 SD above the mean (+ 1 DS).

This significant interaction term indicates that the adverse effect of social media addiction risk on adolescent’s life satisfaction was less pronounced the more they are able to accept and perspective change in front of a stress situation. For example, when acceptance and perspective change is one SD below the mean, the beta regression coefficient *β* = −0.39, (*p* < 0.001) whereas their value is *β* = −0.22, (*p* < 0.001) at the mean value of acceptance and perspective change, and the relationship between social media addiction risk with students’ life satisfaction was not significant when acceptance and perspective change is one SD above the mean, *β* = −0.06, *p* > 0.05 (see simple slopes, Figure 1).

**Figure 2:**
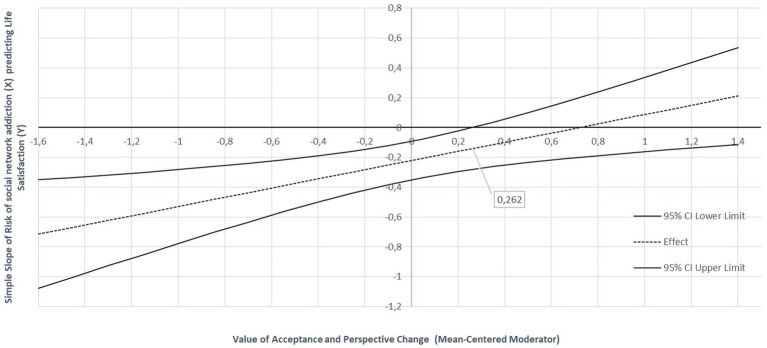
Johnson Neyman graph of the conditional effect of risk of social network addiction on student’s life satisfaction. The Y axis represents the simple slopes effect. The X axis represents the moderator values (Acceptance and Perspective Change coping strategy). Created on bases Johnson Neyman PROCESS macro data.

In fact, the JN technique indicated that when the mean-centered level of acceptance and change perspective is higher than 0.262 (corresponding to 2.70 points on the original scale), there is no correlation between social media addiction risk with students’ life satisfaction (the confidence interval contains the value zero). Below this value it indicates that the adverse effect of social media addiction risk on adolescent’s life satisfaction is less pronounced-or these simple slopes are less negative-as they are more able to accept and change perspective when dealing with a stressful situation (see Figure 2).

## Discussion

Our results support our hypothesis that social media addiction risk negatively correlates to life satisfaction. Also, adaptive strategies such as seeking socio-emotional support, active coping and acceptance, and being able to change perspective were positively correlated to adolescents’ life satisfaction. Moreover, maladaptive strategies and substance use were negatively correlated with life satisfaction. The interaction effect between these variables on life satisfaction was observed only for the acceptance and perspective-taking strategy, albeit with a small effect size.

The results regarding a negative association between the risk of internet and social network addiction with student life satisfaction is similar to that reported in other studies conducted both before and after the pandemic, negatively impacting all areas of life satisfaction (53). For example, one study that sought to comprehend the relationship between internet addiction and areas of life satisfaction found that these variables were negatively correlated in a highly significant way (31). Similarly, another study aimed to work out the effects of uncertainty during the pandemic in a group of adolescents. A negative and highly significant connection between internet addiction and satisfaction with students’ lives was observed (53–55). In turn, as reported by Yu and Shek (15) in their longitudinal study, internet addiction would explain worse outcomes in adolescents’ life satisfaction, which is in keeping with what is reported in the present work, as internet addiction would explain 3.7% of the variance in life satisfaction.

Our second result is consistent with previous research indicating that cognitive and problem-focused coping strategies have been found to mitigate the negative impact of pandemic-related stressors on Western adolescents’ life satisfaction (42) and serve as protective factors against emotional maladjustment for Eastern adolescents (56).

The COVID-19 pandemic can be characterized as a period of high unpredictability and uncertainty that adolescents are experiencing for the first time in their lives (7, 42, 57). Furthermore, it involves a series of unanticipated/manageable events; for example, the possibility of getting sick with COVID-19, the imposition of staying locked inside during quarantines, among others (58). In these cases, the use of adaptive coping strategies to face these stressful events is very relevant in order to maintain better levels of life satisfaction.

It is also relevant to note that coping strategies used by adolescents might be influenced by their parents’ actions and/or family dynamics (59–61). For example, based on bidirectional effects between adults and their children, Shi and Wang (62) reported that adolescents who perceived more supportive parental reactions to their negative emotions before the COVID-19 pandemic used more approach-coping and less avoidance-coping when managing pandemic-related stress, which was associated with lower emotional maladjustment for adolescents during the pandemic. In contrast, those who perceived more unsupportive parental reactions before the pandemic used more avoidance-coping, which was associated with higher emotional maladjustment symptoms. Thus, the coping strategies used by adolescents potentially capture the intrafamilial relational dynamics in which they were immersed during the pandemic that had impacts on adolescents’ life satisfaction (58).

Maladaptive coping strategies may also indirectly impact adolescents’ general life satisfaction. For example, van Loon et al. (63) reported that adolescents with maladaptive coping strategies including self-blame, other-blame, rumination, and catastrophizing before the pandemic experienced higher levels of COVID-19-related concerns about either getting sick themselves or relatives falling ill. The authors conclude that adolescents who use more maladaptive coping strategies in the face of stressful situations may view the risk of becoming seriously ill to be the most unpredictable and uncontrollable. This fear and/or worry has a significant effect in reducing their satisfaction with life (64).

We evaluated a moderation model in which four stressful management strategies, namely, acceptance and perspective-taking, seeking socio-emotional support, active coping, and maladaptive strategies, conditioned the relationship between social media addiction risk and life satisfaction (controlled by demographic variables and the specific strategies of using humor, religion, and substances). It is striking that only the ability to accept and take perspective on situations could buffer the relationship between social network use and life satisfaction.

The COVID-19 pandemic and associated restriction measures were beyond the control of individuals, particularly children and adolescents (58). Acceptance means facing reality even if it does not fit one’s expectations or desires and the willingness to deal with the situation nevertheless. Acceptance can help one adapt to unchangeable adverse events by helping maintain the individual’s psychological wellbeing and capacity to act. Thus, acceptance is characterized as giving up the striving for situation control, withdrawing the individual’s energy from a situation where further attempts would be fruitless, and redirecting the energy into other more constructive actions (65). In this sense, the ability to accept COVID-19 may be a coping strategy that—unlike the others considered in the study—would have a direct effect on the general wellbeing of adolescents.

Moreover, finding a positive aspect of the COVID-19 pandemic (positive reframing) and being able to self-distract are two useful coping strategies, that have the potential to positively influence satisfaction with life. For example, given that adolescents were indoors most of the time, they could have developed domestic or other supportive activities that let them distract or entertain themselves (66, 67). Our interaction effect indicated that the level of life satisfaction was not correlated to the abusive use of social networks for those adolescents who were highly capable of accepting the COVID-19 situation and its restrictions on daily life, found something positive about it (positive reframing) and were able to self-distract. In contrast, adolescents who reported having low or average levels of acceptance and perspective-taking presented a negative correlation between abusive use of social networks and general satisfaction with life, as this relationship was more marked as lower levels of acceptance and perspective-taking of adolescents.

We found that adolescents with lower levels of acceptance were at greater risk of social network addiction the lower their satisfaction with life, which is consistent with other studies that have shown that social network abuse is associated with higher levels of anxiety and depression, increased isolation, decreased physical activity, low self-esteem, poor sleep quality, and more (68). In contrast, when they had higher acceptance levels with positive reframing, social network addiction risk did not influence life satisfaction. In this sense, using this strategy turned them into resilient people. There is consensus that two crucial aspects must be present to qualify as resilient; an experience of adversity and a subsequent positive adaptation (69, 70). Such an adolescent can positively overcome their risky use of social media.

Studies that include mental health problems have reported on this buffer role of psychological flexibility (71, 72). For example, Liu et al. (73) discussed the moderating role of psychological inflexibility in the association between distress-driven impulsivity and problematic internet use. They conclude that people who engage in instantly gratifying behaviors such as internet use/abuse when facing stressful events or negative emotions find short-term relief for their negative mood, thereby possibly incorporating this as a coping strategy through negative reinforcement. Over time, such coping strategies may bring aversive consequences; for example, too much time spent online at the expense of time spent on schooling or increased isolation. At this point, flexible individuals may be able to change their behavior and avoid negative consequences. However, inflexible individuals may be unable to do so and would be more likely to continue using the internet as a coping strategy, despite the consequences (74). So, in the case of our interaction results, when adolescents reported higher levels of acceptance and positive reframing, which reflects higher psychological flexibility, life satisfaction was not influenced by social media addiction risk because they were able to change their risky addiction behavior as they experienced the aversive consequences associated with it. This individual mechanism is an example of resilience theory (75) which highlights the importance of individual resources as protective factors to manage risk, and adverse experiences, such as the Pandemic.

Despite our results, the study has its limitations. The first one is its cross-sectional design, which does not allow inferences about causality between the occurrence of social media addiction risk or coping strategies and adolescents’ life satisfaction. A second limitation is the use of self-reported data without any other sources of information. However, this was the best way to collect data from adolescents during the pandemic period. A third limitation is a factorial solution for the Brief Coping Orientation to Problems Experienced scale. As we reported in the study, there was not only one factorial solution for this measure, which can limit the comparison with other studies using the same instrument. Nevertheless, we reported the factorial structure of the study sample. Lastly, even though we controlled for age and sex in our analysis, we did not include additional sample characteristics that would enable an examination of subgroups, such as sexual diversity, immigrant students, or those belonging to different ethnic groups.

At a theoretical level, the implications of this research are that it provides new knowledge regarding the relationship between coping mechanisms and satisfaction with adolescent life. It also provides important information regarding the effects of Internet addiction on adolescent life and mental health. These findings are relevant in that they allow us to continue to delve deeper, not only in a pandemic or post pandemic context regarding the implications for adolescent wellbeing and mental health, but also as a situation that is always relevant and current for the infant and adolescent population. On the other hand, the practical implications of this research are related to the fact that it provides information that can be useful for public policy makers, school administrators and people who work directly with children and adolescents in that it provides relevant information regarding which coping mechanisms would be most important to strengthen when generating interventions that could help to address mental health problems associated with Internet addiction that adolescents suffer so frequently today. Also, the present work provides relevant information that helps to put the focus and importance of working on promoting mental health and satisfaction with adolescent life especially in this post pandemic time that has greatly and strongly affected this age group.

## Data availability statement

The raw data supporting the conclusions of this article will be made available by the authors, without undue reservation.

## Ethics statement

The studies involving human participants were reviewed and approved by the Universidad del Desarrollo Ethics Committee. Written informed consent to participate in this study was provided by the participants’ legal guardian/next of kin.

## Author contributions

JV and MR-R: conceptualization. JV, JP, MR-R, and JR: methodology. JP, JR, and MR-R: formal analysis. MR-R and MC: data curation. All authors contributed to the manuscript writing of sections of the manuscript, read, and approved the submitted version.

## Funding

This work was funded by the ANID, FONDECYT REGULAR 1201576.

## Conflict of interest

The authors declare that the research was conducted in the absence of any commercial or financial relationships that could be construed as a potential conflict of interest.

## Publisher’s note

All claims expressed in this article are solely those of the authors and do not necessarily represent those of their affiliated organizations, or those of the publisher, the editors and the reviewers. Any product that may be evaluated in this article, or claim that may be made by its manufacturer, is not guaranteed or endorsed by the publisher.
