# Supplementary material for: Wellbeing, social media addiction and coping strategies among Chilean adolescents during the pandemic
Source: Front Psychiatry. 2023 Aug 29;14:1211431. doi: 10.3389/fpsyt.2023.1211431 (PMC10497769; doi:10.3389/fpsyt.2023.1211431)
Supplement: Supplementary file 1 [file Data_Sheet_1.docx]

Supplementary Material: Factorial Analysis of the Brief COPE Scale

Well-being, Internet Addiction and Coping Strategies Among Chilean Adolescents During the Pandemic

Jorge J. Varela^1*^, J. Carola Pérez^1^, Matias E. Rodriguez-Rivas^1^, Josefina Chuecas^1^, Javiera Romo^1^

^1^Facultad de Psicología, Universidad del Desarrollo, Chile

*** Correspondence:**Jorge J. Varela
jovarela@udd.cl

**Factorial Analysis of the Brief COPE Scale**

To determine the factor structure of the COPE scale, an exploratory factor analysis (EFA) and confirmatory factor analysis (CFA) were performed.

The total sample was divided into two subsamples with the R studio *sample* function, which generates a random division of samples, preceded by the *set.seed* function to save the bases with the same subjects. The first subsample for EFA represents 50% of the sample (*n*=660) and the second subsample for CFA represents 50% (*n*=660).

For the EFA we used the *fa* function from the *psych* library, which uses ordinary least squares (OLS) to find the minimum residuals solution (minres) and the generalized weighted least squares (GLS) factorization method and oblimin rotation.

The *cfa* function of the *Lavaan* package was used for the confirmatory factor analysis. Given the ordinal nature of the response alternatives (likert scale from 1 to 4) and the lack of multivariate normality, the model parameters were estimated using the weighted least squares method adjusted for mean and variance (WLSMV).

The Cronbach's alpha statistic and the *cronbach.alpha* function of the *ltm* package were used for the internal consistency analysis.

The statistical program R Studio version 4.1.3 (R Core Team, 2022) was used for all analyses.

**Exploratory factor analysis (EFA)**

To find out how many items the COPE scale is measuring, the parallel analysis function of the psych package was used in the first instance. The analysis suggests that the number of factors to extract is 8 and the number of components is 7; however, it was decided to generate the solution with 7 factors to have greater interpretability according to the theory (substantive meaning of the indicators) and to avoid errors associated with the number of items per factor, since different authors recommend a minimum of 3 or 4 items per factor (Fabrigar et al., 1999; Ferrando and Anguiano-Carrasco, 2010). Specifically, in the 8-factor solution there are two factors with only one item that are highly correlated with each other (common factors) and 4 items need to be removed due to their low factor loading , while in the 7-factor solution only 2 items need to be removed and there are no factors with less than 2 items.

Then, an exploratory factor analysis of 7 factors was performed, which proposed eliminating two items: cop23 (I express my negative feelings (venting)) and cop3 (I accept the reality of what has happened (acceptance)) because they did not load on any factor. In addition, two items were reversed for loading negatively on a factor. Table 1 shows the distribution of the items in the factors and the factor loadings.

**Table 1**

*Results from an Exploratory Factor Analysis of Brief–Cope Scale*

| Factor | Item number | Question (Spanish) | Question (English) | Original Theoretical Factorial Structure (Carver, 1997) | Factor Loadings |
| --- | --- | --- | --- | --- | --- |
| Socio-  Emotional Support | 28 | Consigo que otras personas me ayuden o aconsejen | I’ve been getting help and advice from other people | Instrumental support | 0.84 |
|  | 17 | Consigo el consuelo y la comprensión de alguien | I’ve been getting comfort and understanding from someone | Emotional support | 0.82 |
|  | 9 | Consigo apoyo emocional de otros | I’ve been getting emotional support from others | Emotional support | 0.79 |
|  | 1 | Intento conseguir que alguien me ayude o aconseje sobre qué hacer | I’ve been trying to get advice or help from other people about what to do | Instrumental support | 0.63 |
| Active Coping | 2 | Concentro mis esfuerzos en hacer algo sobre la situación en la que estoy | I’ve been concentrating my efforts on doing something about the situation I’m in. | Active coping | 0.66 |
|  | 6 | Intento proponer una estrategia sobre qué hacer | I’ve been trying to come up with a strategy about what to do | Planning | 0.64 |
|  | 26 | Pienso detenidamente sobre los pasos a seguir | I’ve been thinking hard about what steps to take | Planning | 0.60 |
|  | 10 | Tomo medidas para intentar que la situación mejore | I’ve been taking action to try to make the situation better | Active coping | 0.58 |
|  | 25^a^ | Renuncio al intento de hacer frente al problema | I’ve been giving up the attempt to cope | Behavioral disengagement | -0.48 |
|  | 11^a^ | Renuncio a intentar ocuparme de ello | I’ve been giving up trying to deal with it | Behavioral disengagement | -0.42 |
| Maladaptative strategies | 27 | Me echo la culpa de lo que ha sucedido | I’ve been blaming myself for things that happened | Self-blame | 0.60 |
|  | 8 | Me critico a mí mismo/a | I’ve been criticizing myself | Self blame | 0.55 |
|  | 13 | Me niego a creer que haya sucedido | I’ve been refusing to believe that it has happening | Denial | 0.46 |
|  | 5 | Me digo a mí mismo/a "esto no es real" | I’ve been saying to myself “this isn’t real” | Denial | 0.44 |
|  | 12 | Digo cosas para dar rienda suelta a mis sentimientos desagradables | I’ve been saying things to let my unpleasant feeling escape | Venting | 0.39 |
| Substance Use | 24 | Utilizo alcohol u otras drogas para ayudarme a superarlo | I’ve been using alcohol or other drugs to help me get through it | Substance use | 0.93 |
|  | 15 | Utilizo alcohol u otras drogas para hacerme sentir mejor | I’ve been using alcohol or other drugs to make myself feel better | Substance use | 0.90 |
| Humor | 19 | Me río de la situación | I’ve been making jokes about it | Humor | 0.82 |
|  | 7 | Hago bromas sobre ello | I’ve been making fun of the situation | Humor | 0.82 |
| Religion | 20 | Rezo o medito | I’ve been praying or meditating | Religion | 0.79 |
|  | 16 | Intento hallar consuelo en mi religión o creencias espirituales | I’ve been trying to find comfort in my religion or spiritual beliefs | Religion | 0.78 |
| Acceptance and change of perspective | 18 | Busco algo bueno en lo que está sucediendo | I’ve been looking for something good in what is happening | Positive reframing | 0.54 |
|  | 14 | Intento verlo con otros ojos, para hacer que parezca más positivo | I’ve been trying to see it in a different light, to make it seem more positive | Positive reframing | 0.51 |
|  | 21 | Aprendo a vivir con ello | I’ve been learning to live with it | Acceptance | 0.38 |
|  | 22 | Hago algo para pensar menos en ello, tal como ir al cine o ver la televisión | I’ve been doing something to think about it less, such as going yo movies, watching tv | Self distraction | 0.38 |
|  | 4 | Recurro al trabajo o a otras actividades para apartar las cosas de mi mente | I’ve been to turning to work or other activities to take my mind off things | Self distraction | 0.35 |

*Note.* ^a^items inverted. N=660.

The sum of the factor loadings (sum of all squared values) of the socio-emotional support factor is 2.62, active coping is 2.36, maladaptive strategies is 1.76, substance use is 1.75, humor is 1.55, religion is 1.39 and acceptance and change of perspective is 1.36. On the other hand, the factors are only weakly correlated with each other, as can be seen in Table 2.

**Table 2**

*Correlations between factors of the Brief COPE scale*

| Variable | 1. | 2. | 3. | 4. | 5. | 6. | 7. |
| --- | --- | --- | --- | --- | --- | --- | --- |
| 1.Socio-emotional support | - |  |  |  |  |  |  |
| 2.Active coping | .23 | - |  |  |  |  |  |
| 3. Maladaptive strategies | .01 | -.24 | - |  |  |  |  |
| 4. Substance use | -.11 | -.17 | .15 | - |  |  |  |
| 5. Humor | .05 | .00 | .25 | .12 | - |  |  |
| 6. Religion | .24 | .15 | .03 | -.02 | -.05 | - |  |
| 7.Acceptance and change of perspective | .26 | .29 | -.01 | -.09 | .19 | .23 | - |

**Confirmatory factor analysis (CFA)**

To generate the CFA, the items recommended by the EFA were eliminated (cop3, cop23) and the items that loaded negative (cop11 and cop25) were inverted. In addition, an analysis of the modification indexes was performed to explain the covariance matrix between the items, which suggests adding a relationship between the inverted items to the model because their paraphrasing is very similar.

To evaluate the latter model, different fit indices were used: robust χ2 not significant as an indicator of perfect fit, CFI and TLI greater than or equal to 0.95 as indicators of good fit and between .90 and .95 as acceptable values, an RMSEA value not greater than .08 as an acceptable indicator and less than .05 as a good indicator (at 90% confidence interval), and SRMR value less than .08 as acceptable and less than .05 as good (West et al., 2012).

The CFA result shows that all the estimated coefficients are all significant (p<.001), and the variances are all positive, which is a good indicator. Also, as shown in Table 3, all the fit indicators are acceptable and good, indicating that the factor structure for the Chilean adolescent population is confirmed. The factor loadings are shown in Figure 1.

**Table 3**

*Fit indices in Factorial structure of Brief-Cope Scale*

| Modelo | *x*^2^ (gl) | CFI | TLI | RMSEA [90% CI] | SRMR |
| --- | --- | --- | --- | --- | --- |
| Modelo de 7 Factores | 707.619^***^(277) | .91 | .90 | .05 [.046-.055] | .05 |

*Note*. *x*^2^= chi-square test.CFI= comparative fit index, TLI= Tucker-Lewis index, RMSEA= root mean square error approximation, CI=Confidence interval.

**Figure 1**

*Confirmatory Factor Analysis of Brief–Cope Scale*


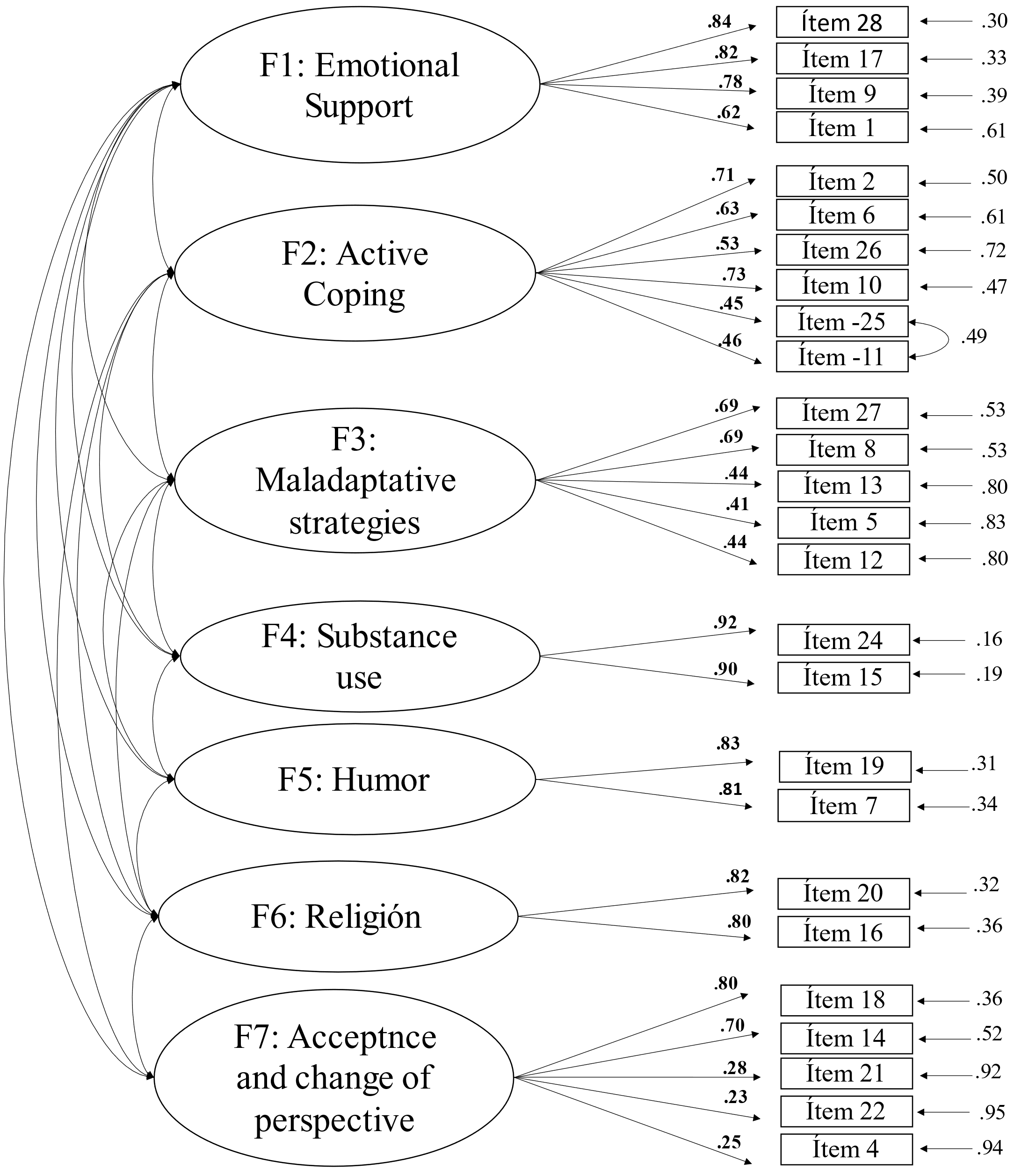


*Note*. Items are numbered in the order presented in Table 1.

Finally, the reliability analysis of the total scale through cronbach's alpha analysis (Cronbach, 1951) is *ɑ=*.77, which indicates an acceptable alpha (Cronbach, 1951). When reviewing the reliability indices for each of the factors, we found good indices (substance use, humor and religion), acceptable indices (active coping) and weak indices (socioemotional support, maladaptive strategies, and acceptance and change of perspective) (See Table 4).

**Table 4**

*Psychometric Properties for Brief-Cope Scale*

| Factor | M | SD | Range | Cronbach’s *ɑ* |
| --- | --- | --- | --- | --- |
| Socioemotional support | 9.44 | 2.46 | 4-16 | .67 |
| Active coping | 17.6 | 3.27 | 8-24 | .77 |
| Maladaptive strategies | 10.36 | 2.83 | 5-20 | .67 |
| Substance use | 2.25 | 0.83 | 2-8 | .91 |
| Humor | 4.19 | 1.91 | 2-8 | .81 |
| Religion | 3.13 | 1.52 | 2-8 | .80 |
| Acceptance and change of perspective | 12.93 | 2.75 | 5-20 | .60 |

References

Campos, M., Iraurgui, J., Páez, D., Velasco, C. (2004). Afrontamiento y regulación emocional de hechos estresantes: Un meta-análisis de 13 estudios. *Boletín de Psicología*, 82, 25-44.

Carver, C.S. (1997). You want to measure coping but your protocol’s is too long: Consider the brief. *International Journal of Behavioral Medicin*e, 4(1), 92-100.

Cronbach, L. J. (1951). Coefficient alpha and the internal structure of test. *Psychometrika*, 16, 297-334.

Fabrigar, L. R., Wegener, D. T., MacCallum, R. C., y Strahan, E. J. (1999). Evaluating the use of exploratory factor analysis in psychological research. *Psychological Method*s, 4(3), 272-299.

Ferrando, P. J. y Anguiano-Carrasco, C. (2010). El análisis factorial como técnica de investigación en psicología. *Papeles del Psicológ*o, 31 (1), 18-33

R Core Team (2022). *R: A language and environment for statistical computing*. R Foundation for Statistical Computing, Vienna, Austria. URL [https://www.R-project.org](https://www.r-project.org/)/

Solberg, M.A,, Gridley, M.K,, Peters, R.M.(2022). The Factor Structure of the Brief Cope: A Systematic Review. *Western Journal of Nursing Research*, 44(6), 612-627. doi:10.1177/01939459211012044

West, S.G., Taylor, A.B., Wu, W. (2012). Model fit and model selection in structural equation modeling. En R.H. Hoyle (Ed.), *Handbook of structural equation modeling* (pp. 209-233). Guilford Press.
